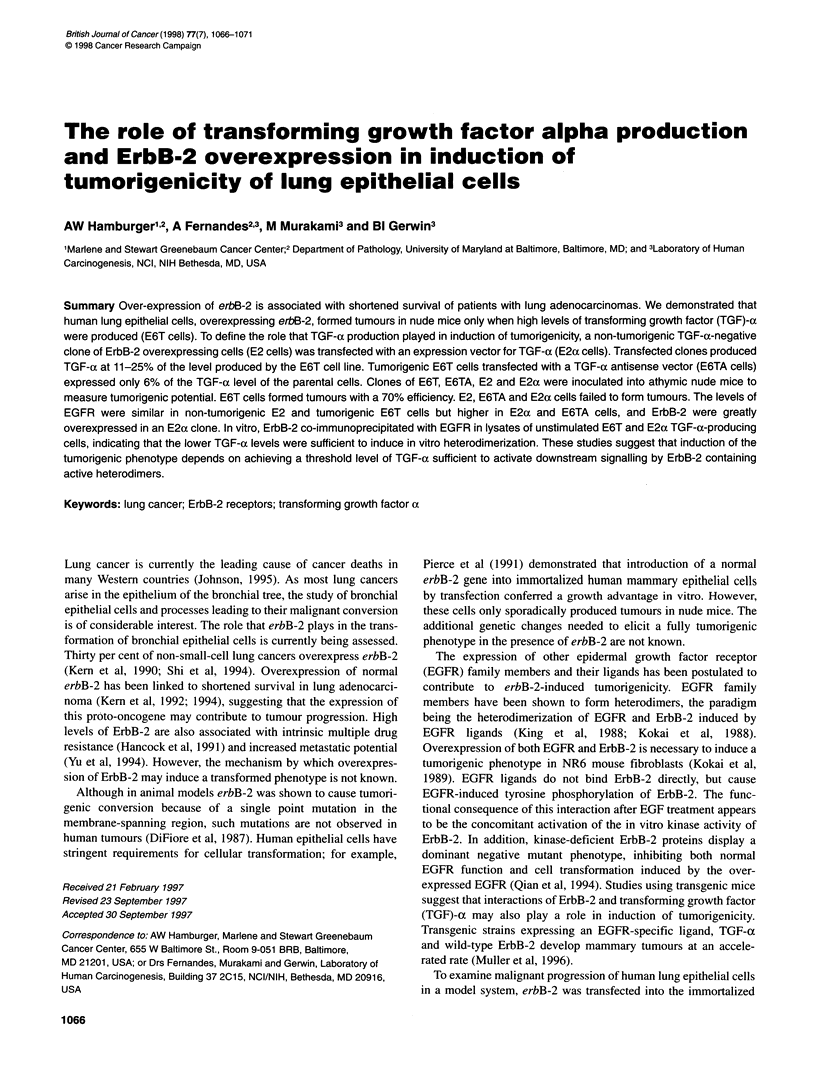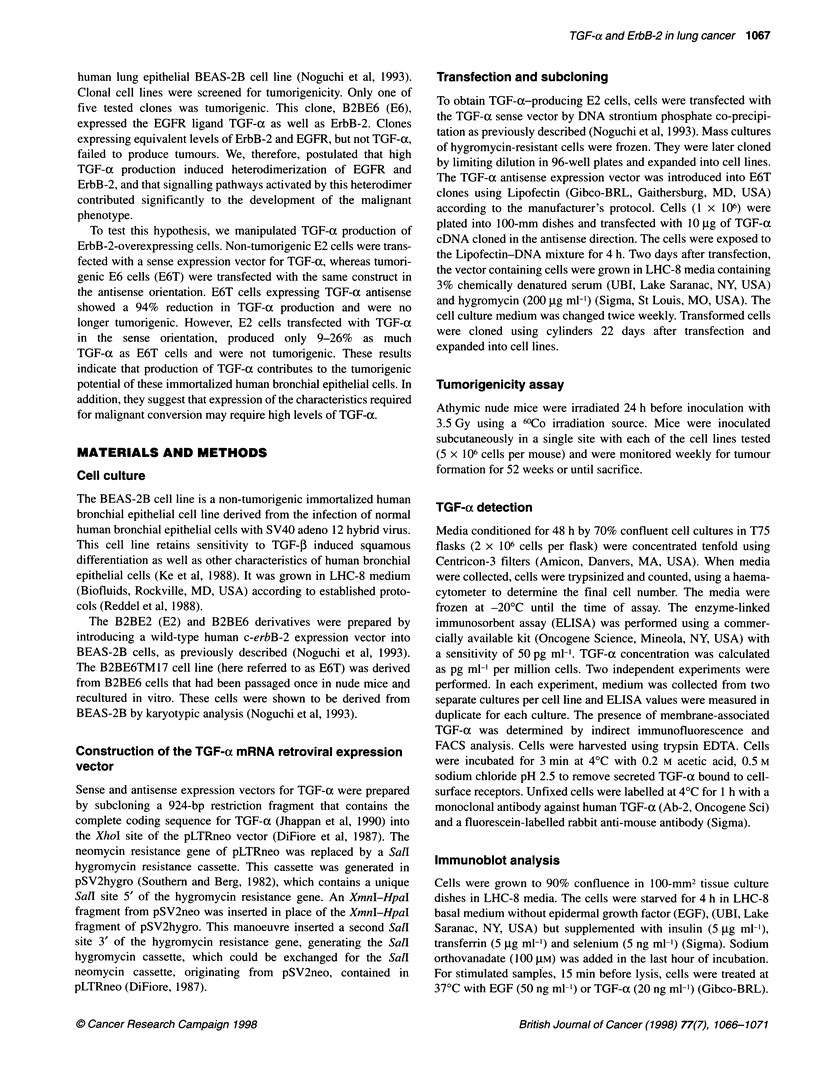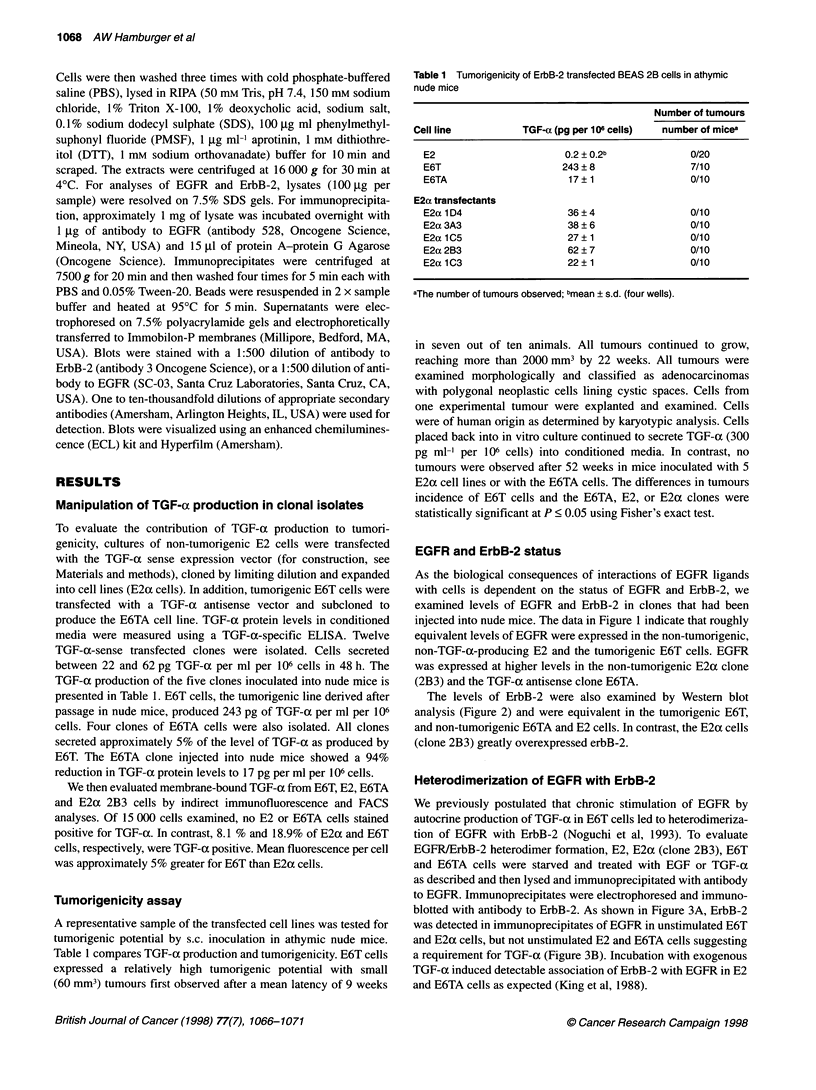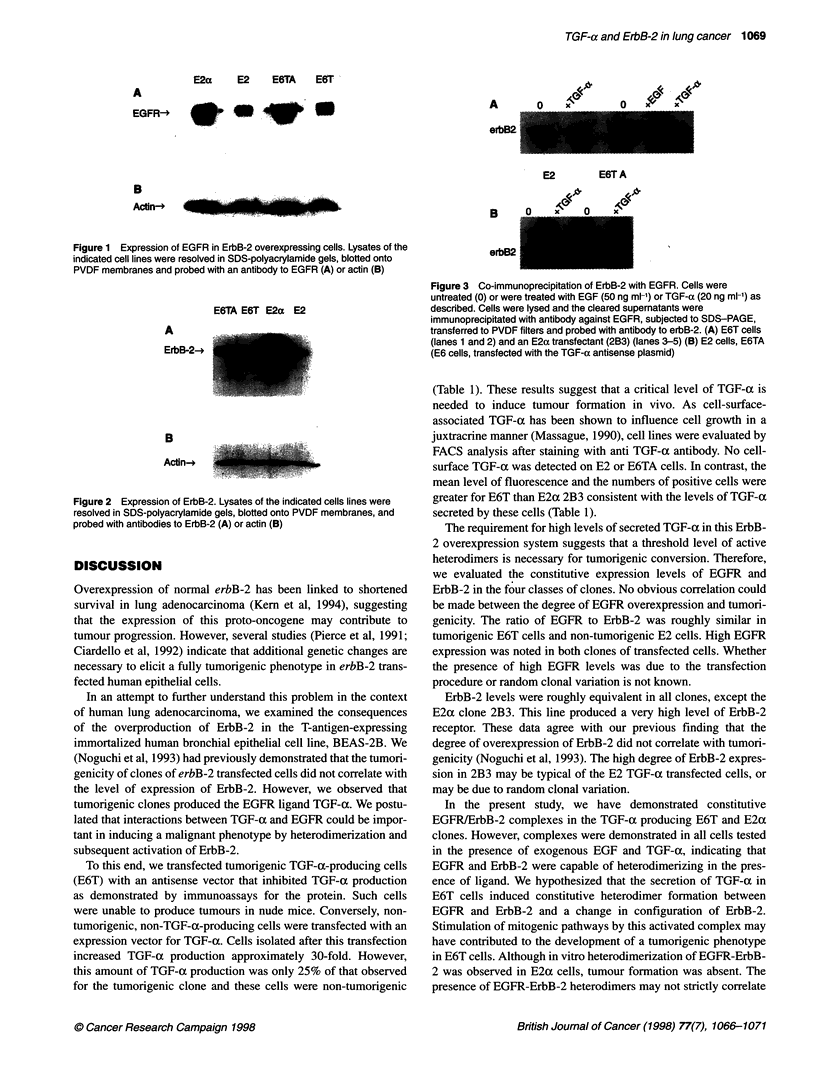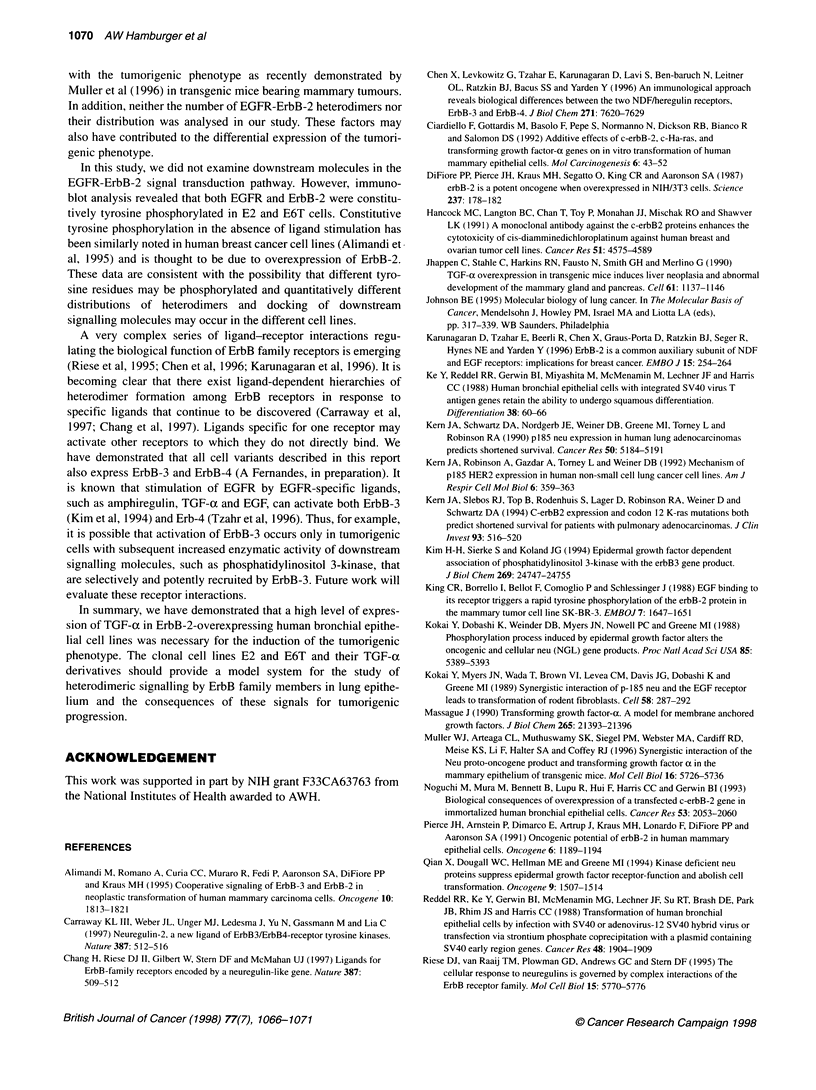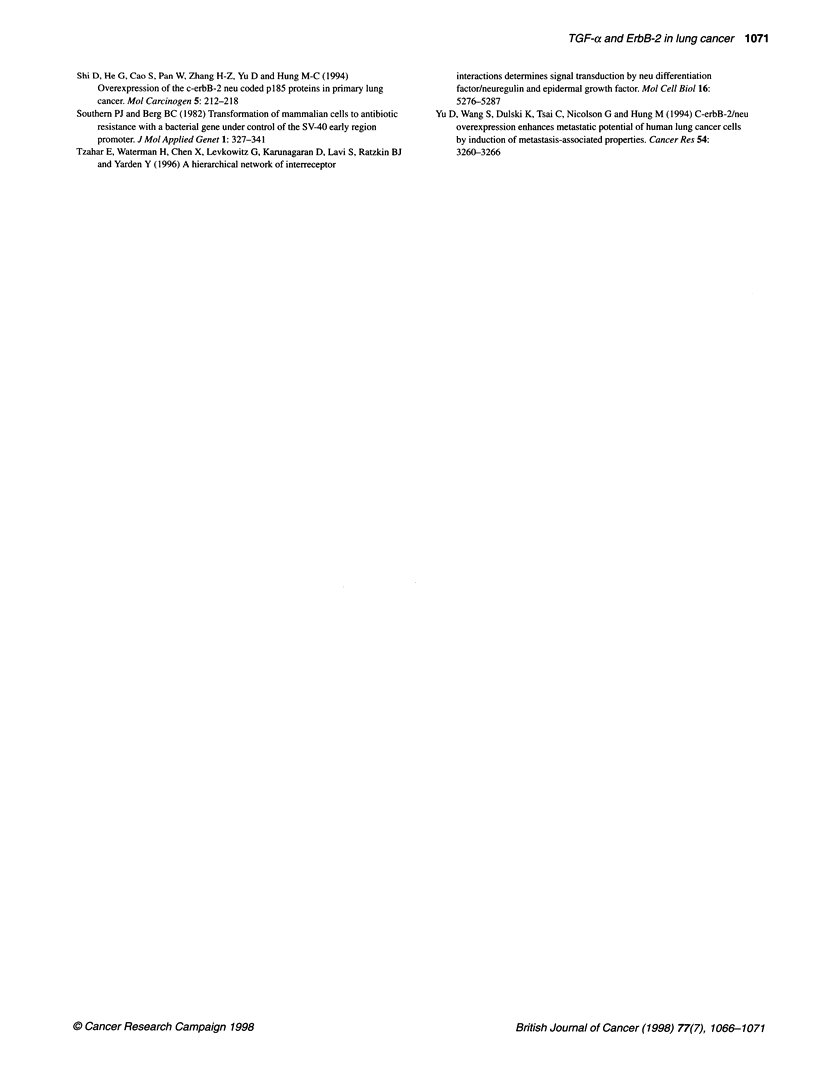# The role of transforming growth factor alpha production and ErbB-2 overexpression in induction of tumorigenicity of lung epithelial cells.

**DOI:** 10.1038/bjc.1998.178

**Published:** 1998-04

**Authors:** A. W. Hamburger, A. Fernandes, M. Murakami, B. I. Gerwin

**Affiliations:** Marlene and Stewart Greenebaum Cancer Center, Department of Pathology, University of Maryland at Baltimore, 21201, USA.

## Abstract

**Images:**


British Journal of Cancer (1998) 77(7), 1066–1071
© 1998 Cancer Research Campaign

### The role of transforming growth factor alpha production and ErbB-2 overexpression in induction of tumorigenicity of lung epithelial cells

**AW Hamburger[1,2], A Fernandes[2,3], M Murakami[3] and BI Gerwin[3]**

[1]Marlene and Stewart Greenebaum Cancer Center;[2] Department of Pathology, University of Maryland at Baltimore, Baltimore, MD; and [3]Laboratory of Human Carcinogenesis, NCI, NIH Bethesda, MD, USA

**Summary** Over-expression of *erb*B-2 is associated with shortened survival of patients with lung adenocarcinomas. We demonstrated that human lung epithelial cells, overexpressing *erb*B-2, formed tumours in nude mice only when high levels of transforming growth factor (TGF)-α were produced (E6T cells). To define the role that TGF-α production played in induction of tumorigenicity, a non-tumorigenic TGF-α-negative clone of ErbB-2 overexpressing cells (E2 cells) was transfected with an expression vector for TGF-α (E2α cells). Transfected clones produced TGF-α at 11–25% of the level produced by the E6T cell line. Tumorigenic E6T cells transfected with a TGF-α antisense vector (E6TA cells) expressed only 6% of the TGF-α level of the parental cells. Clones of E6T, E6TA, E2 and E2α were inoculated into athymic nude mice to measure tumorigenic potential. E6T cells formed tumours with a 70% efficiency. E2, E6TA and E2α cells failed to form tumours. The levels of EGFR were similar in non-tumorigenic E2 and tumorigenic E6T cells but higher in E2α and E6TA cells, and ErbB-2 were greatly overexpressed in an E2α clone. In vitro, ErbB-2 co-immunoprecipitated with EGFR in lysates of unstimulated E6T and E2α TGF-α-producing cells, indicating that the lower TGF-α levels were sufficient to induce in vitro heterodimerization. These studies suggest that induction of the tumorigenic phenotype depends on achieving a threshold level of TGF-α sufficient to activate downstream signalling by ErbB-2 containing active heterodimers.

**Keywords:** lung cancer; ErbB-2 receptors; transforming growth factor α

Lung cancer is currently the leading cause of cancer deaths in many Western countries (Johnson, 1995). As most lung cancers arise in the epithelium of the bronchial tree, the study of bronchial epithelial cells and processes leading to their malignant conversion is of considerable interest. The role that *erb*B-2 plays in the transformation of bronchial epithelial cells is currently being assessed. Thirty per cent of non-small-cell lung cancers overexpress *erb*B-2 (Kern et al, 1990; Shi et al, 1994). Overexpression of normal *erb*B-2 has been linked to shortened survival in lung adenocarcinoma (Kern et al, 1992; 1994), suggesting that the expression of this proto-oncogene may contribute to tumour progression. High levels of ErbB-2 are also associated with intrinsic multiple drug resistance (Hancock et al, 1991) and increased metastatic potential (Yu et al, 1994). However, the mechanism by which overexpression of ErbB-2 may induce a transformed phenotype is not known.

Although in animal models *erb*B-2 was shown to cause tumorigenic conversion because of a single point mutation in the membrane-spanning region, such mutations are not observed in human tumours (DiFiore et al, 1987). Human epithelial cells have stringent requirements for cellular transformation; for example,

*Received 21 February 1997*
*Revised 23 September 1997*
*Accepted 30 September 1997*

*Correspondence to:* AW Hamburger, Marlene and Stewart Greenebaum Cancer Center, 655 W Baltimore St., Room 9-051 BRB, Baltimore, MD 21201, USA; or Drs Fernandes, Murakami and Gerwin, Laboratory of Human Carcinogenesis, Building 37 2C15, NCI/NIH, Bethesda, MD 20916, USA

Pierce et al (1991) demonstrated that introduction of a normal *erb*B-2 gene into immortalized human mammary epithelial cells by transfection conferred a growth advantage in vitro. However, these cells only sporadically produced tumours in nude mice. The additional genetic changes needed to elicit a fully tumorigenic phenotype in the presence of *erb*B-2 are not known.

The expression of other epidermal growth factor receptor (EGFR) family members and their ligands has been postulated to contribute to *erb*B-2-induced tumorigenicity. EGFR family members have been shown to form heterodimers, the paradigm being the heterodimerization of EGFR and ErbB-2 induced by EGFR ligands (King et al, 1988; Kokai et al, 1988). Overexpression of both EGFR and ErbB-2 is necessary to induce a tumorigenic phenotype in NR6 mouse fibroblasts (Kokai et al, 1989). EGFR ligands do not bind ErbB-2 directly, but cause EGFR-induced tyrosine phosphorylation of ErbB-2. The functional consequence of this interaction after EGF treatment appears to be the concomitant activation of the in vitro kinase activity of ErbB-2. In addition, kinase-deficient ErbB-2 proteins display a dominant negative mutant phenotype, inhibiting both normal EGFR function and cell transformation induced by the overexpressed EGFR (Qian et al, 1994). Studies using transgenic mice suggest that interactions of ErbB-2 and transforming growth factor (TGF)-α may also play a role in induction of tumorigenicity. Transgenic strains expressing an EGFR-specific ligand, TGF-α and wild-type ErbB-2 develop mammary tumours at an accelerated rate (Muller et al, 1996).

To examine malignant progression of human lung epithelial cells in a model system, *erb*B-2 was transfected into the immortalized human lung epithelial BEAS-2B cell line (Noguchi et al, 1993). Clonal cell lines were screened for tumorigenicity. Only one of five tested clones was tumorigenic. This clone, B2BE6 (E6), expressed the EGFR ligand TGF-α as well as ErbB-2. Clones expressing equivalent levels of ErbB-2 and EGFR, but not TGF-α, failed to produce tumours. We, therefore, postulated that high TGF-α production induced heterodimerization of EGFR and ErbB-2, and that signalling pathways activated by this heterodimer contributed significantly to the development of the malignant phenotype.

To test this hypothesis, we manipulated TGF-α production of ErbB-2-overexpressing cells. Non-tumorigenic E2 cells were transfected with a sense expression vector for TGF-α, whereas tumorigenic E6 cells (E6T) were transfected with the same construct in the antisense orientation. E6T cells expressing TGF-α antisense showed a 94% reduction in TGF-α production and were no longer tumorigenic. However, E2 cells transfected with TGF-α in the sense orientation, produced only 9–26% as much TGF-α as E6T cells and were not tumorigenic. These results indicate that production of TGF-α contributes to the tumorigenic potential of these immortalized human bronchial epithelial cells. In addition, they suggest that expression of the characteristics required for malignant conversion may require high levels of TGF-α.

### MATERIALS AND METHODS

### Cell culture

The BEAS-2B cell line is a non-tumorigenic immortalized human bronchial epithelial cell line derived from the infection of normal human bronchial epithelial cells with SV40 adeno 12 hybrid virus. This cell line retains sensitivity to TGF-β induced squamous differentiation as well as other characteristics of human bronchial epithelial cells (Ke et al, 1988). It was grown in LHC-8 medium (Biofluids, Rockville, MD, USA) according to established protocols (Reddel et al, 1988).

The B2BE2 (E2) and B2BE6 derivatives were prepared by introducing a wild-type human c-*erb*B-2 expression vector into BEAS-2B cells, as previously described (Noguchi et al, 1993). The B2BE6TM17 cell line (here referred to as E6T) was derived from B2BE6 cells that had been passaged once in nude mice and recultured in vitro. These cells were shown to be derived from BEAS-2B by karyotypic analysis (Noguchi et al, 1993).

### Construction of the TGF-α mRNA retroviral expression vector

Sense and antisense expression vectors for TGF-α were prepared by subcloning a 924-bp restriction fragment that contains the complete coding sequence for TGF-α (Jhappan et al, 1990) into the *Xho*I site of the pLTRneo vector (DiFiore et al, 1987). The neomycin resistance gene of pLTRneo was replaced by a *Sal*I hygromycin resistance cassette. This cassette was generated in pSV2hygro (Southern and Berg, 1982), which contains a unique *Sal*I site 5′ of the hygromycin resistance gene. An *Xmn*I–*Hpa*I fragment from pSV2neo was inserted in place of the *Xmn*I–*Hpa*I fragment of pSV2hygro. This manoeuvre inserted a second *Sal*I site 3′ of the hygromycin resistance gene, generating the *Sal*I hygromycin cassette, which could be exchanged for the *Sal*I neomycin cassette, originating from pSV2neo, contained in pLTRneo (DiFiore, 1987).

### Transfection and subcloning

To obtain TGF-α–producing E2 cells, cells were transfected with the TGF-α sense vector by DNA strontium phosphate co-precipitation as previously described (Noguchi et al, 1993). Mass cultures of hygromycin-resistant cells were frozen. They were later cloned by limiting dilution in 96-well plates and expanded into cell lines. The TGF-α antisense expression vector was introduced into E6T clones using Lipofectin (Gibco-BRL, Gaithersburg, MD, USA) according to the manufacturer's protocol. Cells ($1 \times 10^6$) were plated into 100-mm dishes and transfected with 10 μg of TGF-α cDNA cloned in the antisense direction. The cells were exposed to the Lipofectin–DNA mixture for 4 h. Two days after transfection, the vector containing cells were grown in LHC-8 media containing 3% chemically denatured serum (UBI, Lake Saranac, NY, USA) and hygromycin (200 μg $ml^{-1}$) (Sigma, St Louis, MO, USA). The cell culture medium was changed twice weekly. Transformed cells were cloned using cylinders 22 days after transfection and expanded into cell lines.

### Tumorigenicity assay

Athymic nude mice were irradiated 24 h before inoculation with 3.5 Gy using a $^{60}Co$ irradiation source. Mice were inoculated subcutaneously in a single site with each of the cell lines tested ($5 \times 10^6$ cells per mouse) and were monitored weekly for tumour formation for 52 weeks or until sacrifice.

### TGF-α detection

Media conditioned for 48 h by 70% confluent cell cultures in T75 flasks ($2 \times 10^6$ cells per flask) were concentrated tenfold using Centricon-3 filters (Amicon, Danvers, MA, USA). When media were collected, cells were trypsinized and counted, using a haemacytometer to determine the final cell number. The media were frozen at –20°C until the time of assay. The enzyme-linked immunosorbent assay (ELISA) was performed using a commercially available kit (Oncogene Science, Mineola, NY, USA) with a sensitivity of 50 pg $ml^{-1}$. TGF-α concentration was calculated as pg $ml^{-1}$ per million cells. Two independent experiments were performed. In each experiment, medium was collected from two separate cultures per cell line and ELISA values were measured in duplicate for each culture. The presence of membrane-associated TGF-α was determined by indirect immunofluorescence and FACS analysis. Cells were harvested using trypsin EDTA. Cells were incubated for 3 min at 4°C with 0.2 M acetic acid, 0.5 M sodium chloride pH 2.5 to remove secreted TGF-α bound to cell-surface receptors. Unfixed cells were labelled at 4°C for 1 h with a monoclonal antibody against human TGF-α (Ab-2, Oncogene Sci) and a fluorescein-labelled rabbit anti-mouse antibody (Sigma).

### Immunoblot analysis

Cells were grown to 90% confluence in 100-$mm^2$ tissue culture dishes in LHC-8 media. The cells were starved for 4 h in LHC-8 basal medium without epidermal growth factor (EGF), (UBI, Lake Saranac, NY, USA) but supplemented with insulin (5 μg $ml^{-1}$), transferrin (5 μg $ml^{-1}$) and selenium (5 ng $ml^{-1}$) (Sigma). Sodium orthovanadate (100 μM) was added in the last hour of incubation. For stimulated samples, 15 min before lysis, cells were treated at 37°C with EGF (50 ng $ml^{-1}$) or TGF-α (20 ng $ml^{-1}$) (Gibco-BRL).

© Cancer Research Campaign 1998

Cells were then washed three times with cold phosphate-buffered saline (PBS), lysed in RIPA (50 mM Tris, pH 7.4, 150 mM sodium chloride, 1% Triton X-100, 1% deoxycholic acid, sodium salt, 0.1% sodium dodecyl sulphate (SDS), 100 μg ml phenylmethylsuphonyl fluoride (PMSF), 1 μg $ml^{-1}$ aprotinin, 1 mM dithiothreitol (DTT), 1 mM sodium orthovanadate) buffer for 10 min and scraped. The extracts were centrifuged at 16 000 ***g*** for 30 min at 4°C. For analyses of EGFR and ErbB-2, lysates (100 μg per sample) were resolved on 7.5% SDS gels. For immunoprecipitation, approximately 1 mg of lysate was incubated overnight with 1 μg of antibody to EGFR (antibody 528, Oncogene Science, Mineola, NY, USA) and 15 μl of protein A–protein G Agarose (Oncogene Science). Immunoprecipitates were centrifuged at 7500 ***g*** for 20 min and then washed four times for 5 min each with PBS and 0.05% Tween-20. Beads were resuspended in 2 × sample buffer and heated at 95°C for 5 min. Supernatants were electrophoresed on 7.5% polyacrylamide gels and electrophoretically transferred to Immobilon-P membranes (Millipore, Bedford, MA, USA). Blots were stained with a 1:500 dilution of antibody to ErbB-2 (antibody 3 Oncogene Science), or a 1:500 dilution of antibody to EGFR (SC-03, Santa Cruz Laboratories, Santa Cruz, CA, USA). One to ten-thousandfold dilutions of appropriate secondary antibodies (Amersham, Arlington Heights, IL, USA) were used for detection. Blots were visualized using an enhanced chemiluminescence (ECL) kit and Hyperfilm (Amersham).

### RESULTS

### Manipulation of TGF-α production in clonal isolates

To evaluate the contribution of TGF-α production to tumorigenicity, cultures of non-tumorigenic E2 cells were transfected with the TGF-α sense expression vector (for construction, see Materials and methods), cloned by limiting dilution and expanded into cell lines (E2α cells). In addition, tumorigenic E6T cells were transfected with a TGF-α antisense vector and subcloned to produce the E6TA cell line. TGF-α protein levels in conditioned media were measured using a TGF-α-specific ELISA. Twelve TGF-α-sense transfected clones were isolated. Cells secreted between 22 and 62 pg TGF-α per ml per $10^6$ cells in 48 h. The TGF-α production of the five clones inoculated into nude mice is presented in Table 1. E6T cells, the tumorigenic line derived after passage in nude mice, produced 243 pg of TGF-α per ml per $10^6$ cells. Four clones of E6TA cells were also isolated. All clones secreted approximately 5% of the level of TGF-α as produced by E6T. The E6TA clone injected into nude mice showed a 94% reduction in TGF-α protein levels to 17 pg per ml per $10^6$ cells.

We then evaluated membrane-bound TGF-α from E6T, E2, E6TA and E2α 2B3 cells by indirect immunofluorescence and FACS analyses. Of 15 000 cells examined, no E2 or E6TA cells stained positive for TGF-α. In contrast, 8.1 % and 18.9% of E2α and E6T cells, respectively, were TGF-α positive. Mean fluorescence per cell was approximately 5% greater for E6T than E2α cells.

### Tumorigenicity assay

A representative sample of the transfected cell lines was tested for tumorigenic potential by s.c. inoculation in athymic nude mice. Table 1 compares TGF-α production and tumorigenicity. E6T cells expressed a relatively high tumorigenic potential with small (60 $mm^3$) tumours first observed after a mean latency of 9 weeks in seven out of ten animals. All tumours continued to grow, reaching more than 2000 $mm^3$ by 22 weeks. All tumours were examined morphologically and classified as adenocarcinomas with polygonal neoplastic cells lining cystic spaces. Cells from one experimental tumour were explanted and examined. Cells were of human origin as determined by karyotypic analysis. Cells placed back into in vitro culture continued to secrete TGF-α (300 pg $ml^{-1}$ per $10^6$ cells) into conditioned media. In contrast, no tumours were observed after 52 weeks in mice inoculated with 5 E2α cell lines or with the E6TA cells. The differences in tumours incidence of E6T cells and the E6TA, E2, or E2α clones were statistically significant at $P \leq 0.05$ using Fisher's exact test.

**Table 1** Tumorigenicity of ErbB-2 transfected BEAS 2B cells in athymic nude mice

| Cell line | TGF-α (pg per $10^6$ cells) | Number of tumours / number of mice[a] |
|---|---|---|
| E2 | 0.2 ± 0.2[b] | 0/20 |
| E6T | 243 ± 8 | 7/10 |
| E6TA | 17 ± 1 | 0/10 |
| **E2α transfectants** | | |
| E2α 1D4 | 36 ± 4 | 0/10 |
| E2α 3A3 | 38 ± 6 | 0/10 |
| E2α 1C5 | 27 ± 1 | 0/10 |
| E2α 2B3 | 62 ± 7 | 0/10 |
| E2α 1C3 | 22 ± 1 | 0/10 |

[a]The number of tumours observed; [b]mean ± s.d. (four wells).

### EGFR and ErbB-2 status

As the biological consequences of interactions of EGFR ligands with cells is dependent on the status of EGFR and ErbB-2, we examined levels of EGFR and ErbB-2 in clones that had been injected into nude mice. The data in Figure 1 indicate that roughly equivalent levels of EGFR were expressed in the non-tumorigenic, non-TGF-α-producing E2 and the tumorigenic E6T cells. EGFR was expressed at higher levels in the non-tumorigenic E2α clone (2B3) and the TGF-α antisense clone E6TA.

The levels of ErbB-2 were also examined by Western blot analysis (Figure 2) and were equivalent in the tumorigenic E6T, and non-tumorigenic E6TA and E2 cells. In contrast, the E2α cells (clone 2B3) greatly overexpressed erbB-2.

### Heterodimerization of EGFR with ErbB-2

We previously postulated that chronic stimulation of EGFR by autocrine production of TGF-α in E6T cells led to heterodimerization of EGFR with ErbB-2 (Noguchi et al, 1993). To evaluate EGFR/ErbB-2 heterodimer formation, E2, E2α (clone 2B3), E6T and E6TA cells were starved and treated with EGF or TGF-α as described and then lysed and immunoprecipitated with antibody to EGFR. Immunoprecipitates were electrophoresed and immunoblotted with antibody to ErbB-2. As shown in Figure 3A, ErbB-2 was detected in immunoprecipitates of EGFR in unstimulated E6T and E2α cells, but not unstimulated E2 and E6TA cells suggesting a requirement for TGF-α (Figure 3B). Incubation with exogenous TGF-α induced detectable association of ErbB-2 with EGFR in E2 and E6TA cells as expected (King et al, 1988).

© Cancer Research Campaign 1998

**Figure 1** Expression of EGFR in ErbB-2 overexpressing cells. Lysates of the indicated cell lines were resolved in SDS-polyacrylamide gels, blotted onto PVDF membranes and probed with an antibody to EGFR (**A**) or actin (**B**)

**Figure 2** Expression of ErbB-2. Lysates of the indicated cells lines were resolved in SDS-polyacrylamide gels, blotted onto PVDF membranes, and probed with antibodies to ErbB-2 (**A**) or actin (**B**)

**Figure 3** Co-immunoprecipitation of ErbB-2 with EGFR. Cells were untreated (0) or were treated with EGF (50 ng $ml^{-1}$) or TGF-α (20 ng $ml^{-1}$) as described. Cells were lysed and the cleared supernatants were immunoprecipitated with antibody against EGFR, subjected to SDS–PAGE, transferred to PVDF filters and probed with antibody to erbB-2. (**A**) E6T cells (lanes 1 and 2) and an E2α transfectant (2B3) (lanes 3–5) (**B**) E2 cells, E6TA (E6 cells, transfected with the TGF-α antisense plasmid)

### DISCUSSION

Overexpression of normal *erb*B-2 has been linked to shortened survival in lung adenocarcinoma (Kern et al, 1994), suggesting that the expression of this proto-oncogene may contribute to tumour progression. However, several studies (Pierce et al, 1991; Ciardello et al, 1992) indicate that additional genetic changes are necessary to elicit a fully tumorigenic phenotype in *erb*B-2 transfected human epithelial cells.

In an attempt to further understand this problem in the context of human lung adenocarcinoma, we examined the consequences of the overproduction of ErbB-2 in the T-antigen-expressing immortalized human bronchial epithelial cell line, BEAS-2B. We (Noguchi et al, 1993) had previously demonstrated that the tumorigenicity of clones of *erb*B-2 transfected cells did not correlate with the level of expression of ErbB-2. However, we observed that tumorigenic clones produced the EGFR ligand TGF-α. We postulated that interactions between TGF-α and EGFR could be important in inducing a malignant phenotype by heterodimerization and subsequent activation of ErbB-2.

To this end, we transfected tumorigenic TGF-α-producing cells (E6T) with an antisense vector that inhibited TGF-α production as demonstrated by immunoassays for the protein. Such cells were unable to produce tumours in nude mice. Conversely, non-tumorigenic, non-TGF-α-producing cells were transfected with an expression vector for TGF-α. Cells isolated after this transfection increased TGF-α production approximately 30-fold. However, this amount of TGF-α production was only 25% of that observed for the tumorigenic clone and these cells were non-tumorigenic (Table 1). These results suggest that a critical level of TGF-α is needed to induce tumour formation in vivo. As cell-surface-associated TGF-α has been shown to influence cell growth in a juxtracrine manner (Massague, 1990), cell lines were evaluated by FACS analysis after staining with anti TGF-α antibody. No cell-surface TGF-α was detected on E2 or E6TA cells. In contrast, the mean level of fluorescence and the numbers of positive cells were greater for E6T than E2α 2B3 consistent with the levels of TGF-α secreted by these cells (Table 1).

The requirement for high levels of secreted TGF-α in this ErbB-2 overexpression system suggests that a threshold level of active heterodimers is necessary for tumorigenic conversion. Therefore, we evaluated the constitutive expression levels of EGFR and ErbB-2 in the four classes of clones. No obvious correlation could be made between the degree of EGFR overexpression and tumorigenicity. The ratio of EGFR to ErbB-2 was roughly similar in tumorigenic E6T cells and non-tumorigenic E2 cells. High EGFR expression was noted in both clones of transfected cells. Whether the presence of high EGFR levels was due to the transfection procedure or random clonal variation is not known.

ErbB-2 levels were roughly equivalent in all clones, except the E2α clone 2B3. This line produced a very high level of ErbB-2 receptor. These data agree with our previous finding that the degree of overexpression of ErbB-2 did not correlate with tumorigenicity (Noguchi et al, 1993). The high degree of ErbB-2 expression in 2B3 may be typical of the E2 TGF-α transfected cells, or may be due to random clonal variation.

In the present study, we have demonstrated constitutive EGFR/ErbB-2 complexes in the TGF-α producing E6T and E2α clones. However, complexes were demonstrated in all cells tested in the presence of exogenous EGF and TGF-α, indicating that EGFR and ErbB-2 were capable of heterodimerizing in the presence of ligand. We hypothesized that the secretion of TGF-α in E6T cells induced constitutive heterodimer formation between EGFR and ErbB-2 and a change in configuration of ErbB-2. Stimulation of mitogenic pathways by this activated complex may have contributed to the development of a tumorigenic phenotype in E6T cells. Although in vitro heterodimerization of EGFR-ErbB-2 was observed in E2α cells, tumour formation was absent. The presence of EGFR-ErbB-2 heterodimers may not strictly correlate

© Cancer Research Campaign 1998

with the tumorigenic phenotype as recently demonstrated by Muller et al (1996) in transgenic mice bearing mammary tumours. In addition, neither the number of EGFR-ErbB-2 heterodimers nor their distribution was analysed in our study. These factors may also have contributed to the differential expression of the tumorigenic phenotype.

In this study, we did not examine downstream molecules in the EGFR-ErbB-2 signal transduction pathway. However, immunoblot analysis revealed that both EGFR and ErbB-2 were constitutively tyrosine phosphorylated in E2 and E6T cells. Constitutive tyrosine phosphorylation in the absence of ligand stimulation has been similarly noted in human breast cancer cell lines (Alimandi et al, 1995) and is thought to be due to overexpression of ErbB-2. These data are consistent with the possibility that different tyrosine residues may be phosphorylated and quantitatively different distributions of heterodimers and docking of downstream signalling molecules may occur in the different cell lines.

A very complex series of ligand–receptor interactions regulating the biological function of ErbB family receptors is emerging (Riese et al, 1995; Chen et al, 1996; Karunagaran et al, 1996). It is becoming clear that there exist ligand-dependent hierarchies of heterodimer formation among ErbB receptors in response to specific ligands that continue to be discovered (Carraway et al, 1997; Chang et al, 1997). Ligands specific for one receptor may activate other receptors to which they do not directly bind. We have demonstrated that all cell variants described in this report also express ErbB-3 and ErbB-4 (A Fernandes, in preparation). It is known that stimulation of EGFR by EGFR-specific ligands, such as amphiregulin, TGF-α and EGF, can activate both ErbB-3 (Kim et al, 1994) and Erb-4 (Tzahr et al, 1996). Thus, for example, it is possible that activation of ErbB-3 occurs only in tumorigenic cells with subsequent increased enzymatic activity of downstream signalling molecules, such as phosphatidylinositol 3-kinase, that are selectively and potently recruited by ErbB-3. Future work will evaluate these receptor interactions.

In summary, we have demonstrated that a high level of expression of TGF-α in ErbB-2-overexpressing human bronchial epithelial cell lines was necessary for the induction of the tumorigenic phenotype. The clonal cell lines E2 and E6T and their TGF-α derivatives should provide a model system for the study of heterodimeric signalling by ErbB family members in lung epithelium and the consequences of these signals for tumorigenic progression.

### ACKNOWLEDGEMENT

This work was supported in part by NIH grant F33CA63763 from the National Institutes of Health awarded to AWH.

### REFERENCES

Alimandi M, Romano A, Curia CC, Muraro R, Fedi P, Aaronson SA, DiFiore PP and Kraus MH (1995) Cooperative signaling of ErbB-3 and ErbB-2 in neoplastic transformation of human mammary carcinoma cells. *Oncogene* **10**: 1813–1821

Carraway KL III, Weber JL, Unger MJ, Ledesma J, Yu N, Gassmann M and Lia C (1997) Neuregulin-2, a new ligand of ErbB3/ErbB4-receptor tyrosine kinases. *Nature* **387**: 512–516

Chang H, Riese DJ II, Gilbert W, Stern DF and McMahan UJ (1997) Ligands for ErbB-family receptors encoded by a neuregulin-like gene. *Nature* **387**: 509–512

Chen X, Levkowitz G, Tzahar E, Karunagaran D, Lavi S, Ben-baruch N, Leitner OL, Ratzkin BJ, Bacus SS and Yarden Y (1996) An immunological approach reveals biological differences between the two NDF/heregulin receptors, ErbB-3 and ErbB-4. *J Biol Chem* **271**: 7620–7629

Ciardiello F, Gottardis M, Basolo F, Pepe S, Normanno N, Dickson RB, Bianco R and Salomon DS (1992) Additive effects of c-erbB-2, c-Ha-ras, and transforming growth factor-α genes on in vitro transformation of human mammary epithelial cells. *Mol Carcinogenesis* **6**: 43–52

DiFiore PP, Pierce JH, Kraus MH, Segatto O, King CR and Aaronson SA (1987) erbB-2 is a potent oncogene when overexpressed in NIH/3T3 cells. *Science* **237**: 178–182

Hancock MC, Langton BC, Chan T, Toy P, Monahan JJ, Mischak RO and Shawver LK (1991) A monoclonal antibody against the c-erbB2 proteins enhances the cytotoxicity of cis-diamminedichloroplatinum against human breast and ovarian tumor cell lines. *Cancer Res* **51**: 4575–4589

Jhappen C, Stahle C, Harkins RN, Fausto N, Smith GH and Merlino G (1990) TGF-α overexpression in transgenic mice induces liver neoplasia and abnormal development of the mammary gland and pancreas. *Cell* **61**: 1137–1146

Johnson BE (1995) Molecular biology of lung cancer. In *The Molecular Basis of Cancer*, Mendelsohn J, Howley PM, Israel MA and Liotta LA (eds), pp. 317–339. WB Saunders, Philadelphia

Karunagaran D, Tzahar E, Beerli R, Chen X, Graus-Porta D, Ratzkin BJ, Seger R, Hynes NE and Yarden Y (1996) ErbB-2 is a common auxiliary subunit of NDF and EGF receptors: implications for breast cancer. *EMBO J* **15**: 254–264

Ke Y, Reddel RR, Gerwin BI, Miyashita M, McMenamin M, Lechner JF and Harris CC (1988) Human bronchial epithelial cells with integrated SV40 virus T antigen genes retain the ability to undergo squamous differentiation. *Differentiation* **38**: 60–66

Kern JA, Schwartz DA, Nordgerb JE, Weiner DB, Greene MI, Torney L and Robinson RA (1990) p185 neu expression in human lung adenocarcinomas predicts shortened survival. *Cancer Res* **50**: 5184–5191

Kern JA, Robinson A, Gazdar A, Torney L and Weiner DB (1992) Mechanism of p185 HER2 expression in human non-small cell lung cancer cell lines. *Am J Respir Cell Mol Biol* **6**: 359–363

Kern JA, Slebos RJ, Top B, Rodenhuis S, Lager D, Robinson RA, Weiner D and Schwartz DA (1994) C-erbB2 expression and codon 12 K-ras mutations both predict shortened survival for patients with pulmonary adenocarcinomas. *J Clin Invest* **93**: 516–520

Kim H-H, Sierke S and Koland JG (1994) Epidermal growth factor dependent association of phosphatidylinositol 3-kinase with the erbB3 gene product. *J Biol Chem* **269**: 24747–24755

King CR, Borrello I, Bellot F, Comoglio P and Schlessinger J (1988) EGF binding to its receptor triggers a rapid tyrosine phosphorylation of the erbB-2 protein in the mammary tumor cell line SK-BR-3. *EMBOJ* **7**: 1647–1651

Kokai Y, Dobashi K, Weinder DB, Myers JN, Nowell PC and Greene MI (1988) Phosphorylation process induced by epidermal growth factor alters the oncogenic and cellular neu (NGL) gene products. *Proc Natl Acad Sci USA* **85**: 5389–5393

Kokai Y, Myers JN, Wada T, Brown VI, Levea CM, Davis JG, Dobashi K and Greene MI (1989) Synergistic interaction of p-185 neu and the EGF receptor leads to transformation of rodent fibroblasts. *Cell* **58**: 287–292

Massague J (1990) Transforming growth factor-α. A model for membrane anchored growth factors. *J Biol Chem* **265**: 21393–21396

Muller WJ, Arteaga CL, Muthuswamy SK, Siegel PM, Webster MA, Cardiff RD, Meise KS, Li F, Halter SA and Coffey RJ (1996) Synergistic interaction of the Neu proto-oncogene product and transforming growth factor α in the mammary epithelium of transgenic mice. *Mol Cell Biol* **16**: 5726–5736

Noguchi M, Mura M, Bennett B, Lupu R, Hui F, Harris CC and Gerwin BI (1993) Biological consequences of overexpression of a transfected c-erbB-2 gene in immortalized human bronchial epithelial cells. *Cancer Res* **53**: 2053–2060

Pierce JH, Arnstein P, Dimarco E, Artrup J, Kraus MH, Lonardo F, DiFiore PP and Aaronson SA (1991) Oncogenic potential of erbB-2 in human mammary epithelial cells. *Oncogene* **6**: 1189–1194

Qian X, Dougall WC, Hellman ME and Greene MI (1994) Kinase deficient neu proteins suppress epidermal growth factor receptor-function and abolish cell transformation. *Oncogene* **9**: 1507–1514

Reddel RR, Ke Y, Gerwin BI, McMenamin MG, Lechner JF, Su RT, Brash DE, Park JB, Rhim JS and Harris CC (1988) Transformation of human bronchial epithelial cells by infection with SV40 or adenovirus-12 SV40 hybrid virus or transfection via strontium phosphate coprecipitation with a plasmid containing SV40 early region genes. *Cancer Res* **48**: 1904–1909

Riese DJ, van Raaij TM, Plowman GD, Andrews GC and Stern DF (1995) The cellular response to neuregulins is governed by complex interactions of the ErbB receptor family. *Mol Cell Biol* **15**: 5770–5776

© Cancer Research Campaign 1998

Shi D, He G, Cao S, Pan W, Zhang H-Z, Yu D and Hung M-C (1994) Overexpression of the c-erbB-2 neu coded p185 proteins in primary lung cancer. *Mol Carcinogen* **5**: 212–218

Southern PJ and Berg BC (1982) Transformation of mammalian cells to antibiotic resistance with a bacterial gene under control of the SV-40 early region promoter. *J Mol Applied Genet* **1**: 327–341

Tzahar E, Waterman H, Chen X, Levkowitz G, Karunagaran D, Lavi S, Ratzkin BJ and Yarden Y (1996) A hierarchical network of interreceptor interactions determines signal transduction by neu differentiation factor/neuregulin and epidermal growth factor. *Mol Cell Biol* **16**: 5276–5287

Yu D, Wang S, Dulski K, Tsai C, Nicolson G and Hung M (1994) C-erbB-2/neu overexpression enhances metastatic potential of human lung cancer cells by induction of metastasis-associated properties. *Cancer Res* **54**: 3260–3266

© Cancer Research Campaign 1998